# Research Progress on Plant Shaker K^+^ Channels

**DOI:** 10.3390/plants13101423

**Published:** 2024-05-20

**Authors:** Guang Yuan, Tongjia Nong, Oluwaseyi Setonji Hunpatin, Chuhan Shi, Xiaoqing Su, Qian Wang, Haobao Liu, Peigang Dai, Yang Ning

**Affiliations:** 1 Tobacco Research Institute, Chinese Academy of Agricultural Sciences, Qingdao 266101, China; 2 Graduate School of Chinese Academy of Agricultural Sciences, Beijing 100081, China

**Keywords:** Shaker K^+^ channel, protein structure, function, regulatory mechanism, research methods

## Abstract

Plant growth and development are driven by intricate processes, with the cell membrane serving as a crucial interface between cells and their external environment. Maintaining balance and signal transduction across the cell membrane is essential for cellular stability and a host of life processes. Ion channels play a critical role in regulating intracellular ion concentrations and potentials. Among these, K^+^ channels on plant cell membranes are of paramount importance. The research of Shaker K^+^ channels has become a paradigm in the study of plant ion channels. This study offers a comprehensive overview of advancements in Shaker K^+^ channels, including insights into protein structure, function, regulatory mechanisms, and research techniques. Investigating Shaker K^+^ channels has enhanced our understanding of the regulatory mechanisms governing ion absorption and transport in plant cells. This knowledge offers invaluable guidance for enhancing crop yields and improving resistance to environmental stressors. Moreover, an extensive review of research methodologies in Shaker K^+^ channel studies provides essential reference solutions for researchers, promoting further advancements in ion channel research.

## 1. Introduction

The cell membrane, the fundamental boundary of life, serves as the structural foundation for precise biological regulation and intricate interactions within organisms. Serving not only as a barrier separating cells from their environment, the cell membrane also facilitates the exchange of information and substances [1]. Key components of the cell membrane, membrane proteins are crucial in facilitating the precise transport of substances and regulating signal transduction across intracellular and extracellular environments. They play an essential role in maintaining intracellular environmental stability, enhancing intercellular communication, and regulating biological processes [2]. Within these complex regulatory mechanisms, ion channels are crucial for transmitting substance information and facilitating organism metabolism. They are foundational for maintaining normal cellular functions and ensuring organismic homeostasis [3].

Among the vast array of ion channels, plant K^+^ channels stand out for their significance. K^+^, crucial for plant growth and development, is regulated in its absorption and transport by plant K^+^ channels, playing a decisive role [4]. Not only do these channels maintain the charge balance between intracellular and extracellular environments, they also contribute to cell-volume regulation, substance transport, and cell metabolism [5].

Shaker K^+^ channels have attracted significant attention for their fundamental role in the regulation of intracellular K^+^ concentrations. These channels maintain cellular electrophysiological balance and play multifaceted roles in plant physiology and environmental adaptability [6]. In conditions like salt stress and drought, plant K^+^ channels regulate ion balance to maintain normal physiological states [7,8].

Consequently, a detailed examination of the function and regulatory mechanisms of plant Shaker K^+^ channels is vital not only for deciphering the molecular underpinnings of plant growth and development but also for offering innovative approaches to enhance crop stress resilience and yield. This review primarily explores the origin, structure, function, regulatory pathways, and identification methods of plant Shaker K^+^ channels. It delves into the molecular regulatory mechanisms of Shaker K^+^ channels, including their screening and identification, offering insights for developing crop varieties that efficiently absorb and utilize K^+^.

## 2. Study History of Shaker K^+^ Channel

### 2.1. The Initial Discovery of Shaker K^+^ Channels

In 1977, Jan and colleagues reported that fruit flies carrying a mutant gene demonstrated leg shaking when anesthetized (first mention of Shaker). Similar leg shaking occurred in wild-type fruit flies upon the use of K^+^ channel blockers. Experiments that monitored neuromuscular signals and conducted electrophysiological recordings led to the hypothesis that this gene mutation impaired K^+^ channel functionality in nerve terminal membranes, causing abnormal neuromuscular transmission [9]. This study highlighted the critical role of K^+^ channels in neuromuscular transmission, signifying the beginning of research into K^+^ channels.

### 2.2. Cloning of the Shaker K^+^ Channel Genes

**Figure 1:**
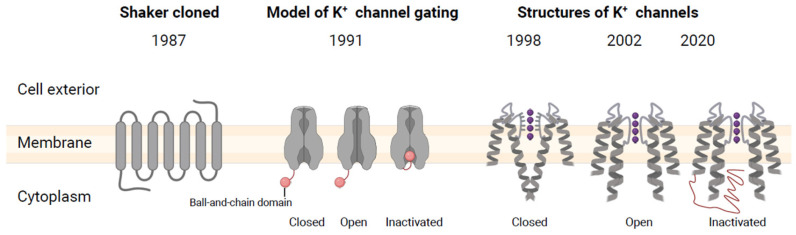
Milestones in the discovery of K^+^ channels [18]. The gray ribbon structure is the channel subunit, the purple balls represent K^+^ ions in the channel, and the orange ribbon represents the ball–chain domain, which blocks the pore of the open but inactive gate.

In 1987, the Tanouye and Jan teams reported the cloning of the Shaker gene from fruit flies nearly simultaneously, utilizing chromosome-walking technology. Hydropathy plot analysis led them to predict that the protein structure contained seven transmembrane regions, later revised to six (S1–S6). Expression of this protein in *Xenopus oocytes* produces rapidly inactivating K^+^ currents, mirroring the electrical signals previously observed in Drosophila muscles [10,11,12]. In 1991, mRNA coexpression experiments suggested for the first time that K^+^ channels were tetramers encircling a central pore (in Figure 1, only two subunits are used to emphasize potassium ion conduction pores. These channels can adopt closed, open, or inactivated conformations, endowed by the “spherical chain” domain at the amino end, which blocks the pores to inactivate the channels), offering initial insights into the protein regions responsible for voltage sensing, inactivation, and selectivity. Following this, a variety of K^+^ channel encoding genes were identified and studied by finding sequences akin to the Shaker gene.

### 2.3. Cloning of the First Plant Shaker K^+^ Channel Gene

The Shaker gene was the first K^+^ channel identified in plants. In 1992, through a method involving the screening of K^+^ uptake-deficient yeast strains and an Arabidopsis cDNA library, two Shaker family members, KAT1 and AKT1, were cloned from Arabidopsis [13,14]. These channels were identified as inward-rectifying K^+^ channels. Subsequent research confirmed that KAT1 and AKT1 share a structural similarity with previously identified animal K^+^ channels, including an S1–S6 transmembrane structure, a pore located between S5 and S6, and a cyclic nucleotide-binding domain (CNBD) that activates the channel [15].

### 2.4. Visualization of Shaker K^+^ Channel Protein Structures

In 1998, Declan A. Doyle and colleagues utilized X-ray crystallography to unveil the crystal structure of the KcsA K^+^ channel in Streptomyces lividans. This breakthrough allowed for the visualization of distinctive features of the Streptomyces lividans K^+^ channel, including four identical subunits that form a cone-shaped structure with a central pore. This pore features carbonyl oxygen atoms tailored for coordinating K^+^. Below the filter, there is a large water-filled cavity and helix dipoles situated at the membrane’s center. The specific orientation of the helix dipoles helps overcome electrostatic instability as ions pass through the membrane’s center, facilitating efficient and selective K^+^ conduction [16]. The advent of cryo-electron microscopy in 2013 marked a significant advancement in ion-channel research, revealing the high-resolution three-dimensional structures of protein complexes in their natural states, including the open, closed, and inactivated states. These structural insights are crucial for understanding channel responses to biochemical signals and the regulation of K^+^ flow within cells [17].

## 3. Shaker K^+^ Channel Protein Structure Characteristics and K^+^ Uptake Mechanisms

### 3.1. Structural Features

The Shaker K^+^ channel is identified as a transmembrane protein located within the plasma membranes of plant cells, as well as in various intracellular membranes such as those of the vacuoles and other organelles [19]. This protein consists of six transmembrane segments (S1–S6), with a pore (P-loop) domain located between S5 and S6. The P-loop domain comprises multiple segments that are embedded within the cell membrane, thereby forming the channel pore. The N-terminus and C-terminus are both situated within the cytoplasm. The N-terminus features a shorter domain comprised of roughly 60 amino acids. In contrast, the C-terminus, located at the conclusion of the sixth transmembrane segment, contains a C-linker with approximately 80 amino acid residues, a cyclic nucleotide-binding domain (CNBD), an ankyrin-related domain (ANKY), and a KHA domain abundant in hydrophobic acidic residues [20].

### 3.2. Voltage Sensing and Channel Opening Mechanism

**Figure 2:**
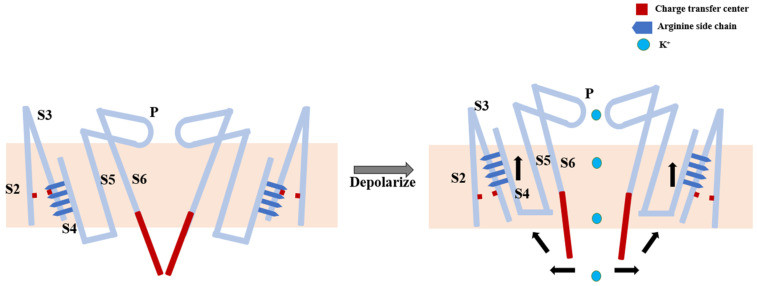
Schematic diagram of the voltage-gating mechanism of voltage-gated K^+^ channels. The red square represents the charge-transfer center, the deep-blue square indicates the position of the amino acid side chains, blue circles represent K^+^, and black arrows represent the direction of pulling.

Segments S1 to S4 are known as voltage-sensing domains (VSD), with S4 playing a pivotal role within the VSD. S5 and S6 form the core of the ion channel. The VSD, sensing changes in membrane potential, encircles the pore domain. S4 contains numerous positively charged amino acid residues (primarily arginines), which are highly conserved and arranged in a regular pattern, with one arginine appearing approximately every three amino acid residues. Structural analyses have shown that, upon membrane potential depolarization, the electric field propels the positively charged arginines on S4 to sequentially move linearly through a charge-transfer center. The vertical upward movement of S4 exerts a rotational pull on the S4–S5 linker. The strong interaction between the S4–S5 linker and the carboxyl-terminal end of S6 leads to the outward bending of the carboxyl-terminal end of S6 due to the outward rotation of the S4–S5 linker, culminating in channel opening (Figure 2) [21].

### 3.3. Selectivity Filter and Permeability

S1 to S4 function as voltage-sensing structures, while S5 and S6 establish the ion channel’s core, exhibiting selectivity for K^+^, Rb^+^, and Cs^+^ ions. However, their permeability to smaller alkali metals like Na^+^ and Li^+^ remains significantly low. Within the K^+^ channel’s selectivity filter, four instances of the TVGYG motif construct a lengthy and narrow passage, creating four K^+^ binding sites at the channel’s center. The carbonyl oxygens on the main chain of the TVGYG motif’s amino acid residues orient towards the channel’s center, with each K^+^ being coordinated by eight surrounding oxygen atoms [22]. The distance from the K^+^ to the oxygen atoms is approximately 0.3 nm. K^+^ traverses the selectivity filter in a dehydrated state. Crystallographic titration experiments reveal two high-affinity K^+^ binding sites within the K^+^ channel’s selectivity filter. Even at low concentrations, K^+^ occupies these high-affinity sites, thereby blocking the entry and permeability of sodium ions [23]. In summary, the narrow and multi-site nature of the K^+^ channel’s selectivity filter ensures selective permeability for K^+^. Sodium ions, with their smaller dehydration radius and shorter ion coordination distance, require more energy for complete dehydration, hindering their ability to compete with K^+^ through the selectivity filter.

### 3.4. Comparison among Shaker and Other K^+^ Channel Families

Members of the Shaker family feature six transmembrane segments with voltage-sensing structures, in contrast to other K^+^ channels that have two to six transmembrane segments but lack voltage-sensing capabilities. The TPK and Kir-like families possess an EF-hand structure at the C-terminus, unable to sense changes in membrane potential but can be directly regulated by Ca^2+^, a feature absent in the Shaker family [24]. Both the Shaker family and other K^+^ channels share a P-loop structure between S5 and S6, while the TPK family features two P-loop structures. Unlike many other K^+^ channels, most Shaker-type K^+^ channel families possess ankyrin regions, which facilitate the connection of K^+^ channels to the cytoskeleton and regulate the cytosol to enhance protein–protein interactions [25]. In conclusion, plant Shaker K^+^ channels exhibit a range of unique characteristics and properties at the protein level, including conserved P-loops and transmembrane regions, voltage sensitivity, and subtype diversity. These differences in structure and function compared to other plant K^+^ channels have significant implications for plant growth, development, and environmental adaptability (see Table 1).

## 4. Advances in Function of Plant Shaker K^+^ Channels

The Shaker K^+^ channel family is the most extensively studied among plant K^+^ channels, playing pivotal roles in plant physiological processes. Arabidopsis thaliana, serving as a model for K^+^ channel research, has yielded comprehensive insights into the functions of various Shaker family members. Research on Arabidopsis offers deep insights into the physiological regulation of Shaker K^+^ channels, laying a crucial foundation for understanding essential processes in plant growth, development, and environmental adaptation. The Arabidopsis Shaker K^+^ channel family comprises nine members, divided into five subfamilies (Groups I to V) based on sequence similarity, gene structure, and functional distinctions. Members of different subfamilies display varied voltage-dependence and rectification properties [26]. Groups I and II consist of hyperpolarization-activated inward rectifier K^+^ channels, mediating K^+^ absorption; Group I features a C-terminal ankyrin region. Group III includes weakly rectified K^+^ channels for both absorption and release. Group IV acts as the regulatory subunit for inward rectifier K^+^ channels. Group V comprises depolarization-activated outward rectifier K^+^ channels, enabling K^+^ release.

### 4.1. Group I

Group I includes three members: AKT1, AKT5, and SPIK (AKT6). AKT1, the first reported Shaker K^+^ channel in plants, is primarily expressed in the epidermis and cortex cells of Arabidopsis roots [27,28]. In Arabidopsis akt1 mutants, where the AKT1 gene is disrupted, the roots show reduced K^+^ uptake capacity, leading to leaf yellowing and loss of greenness under low K^+^ conditions. Electrophysiological measurements reveal that AKT1 encodes an inward-rectifying K^+^ channel, facilitating K^+^ uptake even at low concentrations [29]. SPIK (Shaker pollen inward K^+^ channel) encodes another inward K^+^ channel, primarily expressed in Arabidopsis pollen and pollen tubes. SPIK mutant pollen tubes show significantly reduced inward K^+^ currents, resulting in inhibited growth and decreased pollen competitiveness [30]. AKT5, mainly expressed in Arabidopsis flowers, has a function that remains unclear [31].

### 4.2. Group II

Group II comprises inward K^+^ channels KAT1 and KAT2, located in the guard cells of Arabidopsis. They play a critical role in modulating guard-cell turgor via inward K^+^ flux, thereby regulating stomatal dynamics—opening and closure [32,33]. Studies using heterologous expression in Xenopus oocytes and patch-clamp techniques have shown that Arabidopsis guard cells’ inward Shaker channels primarily form heterotetramers of KAT1 and KAT2, favoring heterotetramer formation over homotetramers. These heterotetramers, made up of KAT1 and KAT2, exhibit unique channel characteristics and subcellular locations compared to homotetramers, enabling the stomata to adapt to external environmental changes [34].

### 4.3. Group III

Group III consists of a single entity, the weakly rectifying K^+^ channel AKT2, identified in Arabidopsis in 1995 [35]. AKT2 plays a crucial role in mediating bidirectional K^+^ flux, which is vital for K^+^ translocation within the Arabidopsis phloem, and thus, it is essential for the plant’s long-distance K^+^ transport [31,36]. Additionally, AKT2 can mediate the co-transport of sucrose and H^+^ [37].

Whole-cell and single-channel voltage clamp data have shown that AKT2 channels exhibit two distinct gating modes. Mode 1 operates at voltages below +100 mV and resembles inward-rectifying K^+^ channels, while Mode 2, less sensitive to voltages below +100 mV, keeps channels perpetually open across the physiological voltage spectrum, enabling bidirectional K^+^ movement [38]. This indicates significant functional plasticity in AKT2, enabling it to fulfill diverse roles in source and sink organ tissues.

### 4.4. Group IV

Group IV is distinguished by a single member, the inward Shaker K^+^ channel regulatory subunit AtKC1. Primarily expressed in root hairs and endodermis; AtKC1’s function goes beyond simple K^+^ conduction. AtKC1 knockout mutants, capable of conducting inward K^+^ currents, show significant changes in gating properties and cation selectivity. This suggests that AtKC1 is crucial for assembling functional K^+^ uptake channels in root hairs, not by directly transporting K^+^ ions but through forming heteromeric complexes with other inward Shaker K^+^ channel subunits, enhancing K^+^ uptake efficiency in Arabidopsis roots [39]. In Arabidopsis root epidermal cells, AtKC1 partners with AKT1 channel subunits to form heteromeric channels. These channels show increased sensitivity to low external K^+^ concentrations, mitigating K^+^ loss more effectively than channels composed solely of AKT1 subunits [40]. Further studies have shown that AtKC1 can also heteromerize with subunits from other inward-rectifying K^+^ channels, like KAT1, KAT2, and AKT2, playing a similar role in preventing K^+^ depletion [41]. Moreover, AtKC1 subunits play a key role in modifying the pore architecture and introducing specific voltage-dependent dynamics to the channel complex. This involves recruiting additional proteins that change the channel’s selectivity profile [42,43,44]. These complex processes enable root epidermal cells to efficiently absorb K^+^ through AKT1/AtKC1 heteromers, thus protecting the cells against cytoplasmic K^+^ loss in K^+^-deficient environments. This mechanism greatly enhances Arabidopsis’s resilience to low K^+^ condition [44].

### 4.5. Group V

Group V consists of two outward rectifier K^+^ channels: GORK and SKOR. In 1998, the SKOR (stelar K^+^ outward rectifier) channel was cloned from Arabidopsis’s pericycle tissue. Using the Xenopus laevis oocyte heterologous system, SKOR was identified as the first plant K^+^ channel with outward rectifier properties [45]. Localized in the stelar cortex and pericycle cells of roots, SKOR knockout mutants show diminished K^+^ content and lower K^+^ concentrations in the xylem sap, highlighting its critical role in mediating K^+^ release into the xylem.

**Figure 3:**
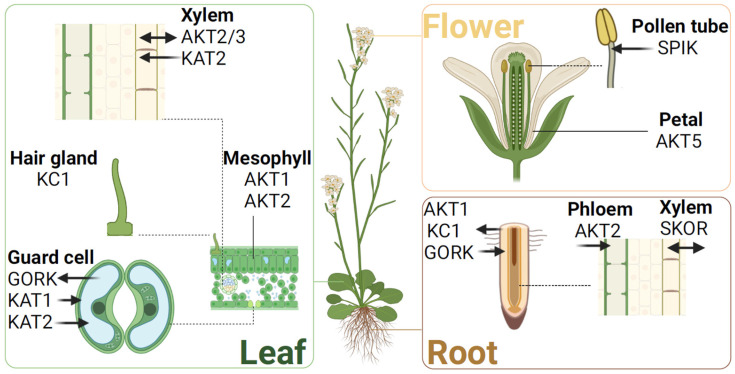
Expression pattern of the Arabidopsis Shaker K^+^ channel.

Following SKOR’s discovery, the GORK channel (guard-cell outward rectifying K^+^ channel) was identified in Arabidopsis guard cells, expanding the known repertoire of plant K^+^ channels with outward rectification capabilities [46]. Dominant-negative GORK mutants in Arabidopsis show significantly reduced outward rectifying K^+^ channel activity, leading to compromised stomatal closure. The complete loss of outward rectifying K^+^ channel activity in GORK knockout mutants suggests that GORK may be the exclusive channel for K^+^ efflux in guard cells, a critical regulator of stomatal function [47]. Additionally, GORK’s presence in root-hair cells highlights its complex role as a K^+^ sensor, involved in modulating both osmotic and membrane potentials [48] (see Figure 3).

## 5. Regulation of Shaker K^+^ Channels

Regulation of Shaker K^+^ channel activity occurs at two primary levels: transcriptional and translational. Transcriptional regulation entails gene activation, transcription factor binding, and modulation of RNA polymerase activity. Conversely, translational regulation covers mRNA stability, translation efficiency, and post-translational modifications, collectively determining a gene’s final expression level and function. Plants uptake K^+^ ions from their environment through K^+^ ion channels and transporter proteins. To precisely regulate K^+^ ion channels, plants have evolved multiple mechanisms to modulate their expression and activity, meeting the physiological needs of different plant parts, stages, and growth environments.

### 5.1. Regulation at Transcriptional Level

Transcriptional regulation is a crucial aspect of controlling eukaryotic gene expression. Low K^+^ stress induces the expression of certain genes coding for K^+^ transporter proteins in plants and also influences the expression of genes related to K^+^ ion channels. Within the Shaker family, Arabidopsis AtKC1 [49], wheat TaAKT1 [50], and maize ZMK1 [51] are induced by low K^+^ levels. OsAKT1 expression is suppressed under salt stress [52].

### 5.2. Post-Translational Regulation

Shaker K^+^ channels possess multiple transmembrane domains, rendering them structurally complex and posing challenges to their translation and folding. Plants utilize post-translational regulation mechanisms to rapidly modulate K^+^ channel activity in response to low K^+^ levels or other environmental stressors.

#### 5.2.1. Regulation by Regulatory Proteins

Phosphorylation represents the predominant regulatory mechanism for Shaker K^+^ channels, extensively studied for its regulation by CBL/CIPK complexes. Calcineurin B-like proteins (CBL), calcium sensors, target a class of protein kinases called CBL-interacting protein kinases (CIPKs). This phosphorylation regulation plays a crucial role throughout the organism [53]. In Arabidopsis, K^+^ deficiency triggers a rapid increase in root Ca2^+^ levels [54]. CBL1/9 binds to CIPK23, accumulating on the plasma membrane, where CIPK23, once activated, phosphorylates and activates AKT1, thereby enhancing K^+^ uptake and transport [55]. Recent studies have refined this pathway, showing plants first activate CBL2/3 in response to low K^+^ signals, which then form complexes with CIPK23 to phosphorylate and regulate AKT1. Concurrently, the phosphatases HAB1/ABI1/ABI2/PP2CA negatively regulate CBL2/3 after K^+^ repletion, impacting the plant’s K^+^ response [56]. Additionally, CIPK1/16, alongside CBL1, can activate AKT1 [57], while phosphorylation by CBL4-CIPK6 regulates AKT2 activation [58]. As confirmed by yeast two-hybrid assays, CIPK25 interacts with AKT1, and both cipk25 and AKT1 mutants exhibit similar developmental defects under waterlogging conditions [59].

Calcium-dependent protein kinases (CDPK) of the Ser/Thr protein kinase family activate upon Ca2^+^ binding, initiating downstream reactions [60]. CPK3, CPK6, and CPK21 phosphorylate to inhibit KAT1 channel activity [61,62]. Specifically expressed in pollen tubes, the Shaker K^+^ channel SPIK is a downstream target of the CPK11-CPK24 pathway, regulating Arabidopsis pollen-tube growth [63]. CPK13 inhibits KAT2 and KAT1 channels in guard cells, reducing stomatal aperture [64], whereas CPK6 enhances KAT2 activity to maintain leaf K^+^ homeostasis [65].

In addition to phosphorylation by CBL1/CIPK23 and dephosphorylation by AIP, AKT1 also directly interacts with CBL10, as demonstrated in yeast two-hybrid, BIFC, and co-immunoprecipitation assays, inhibiting the inward K^+^ current it mediates. Yeast two-hybrid assays indicate CBL10 may compete with CIPK23 for AKT1 binding, thus negatively regulating its activity [66]. In ost1 mutants, the absence of an inward K^+^ current in guard-cell protoplasts suggests KAT1 activation through phosphorylation by OST1 (SnRK2.6) [67]. AKT2’s inward current is reduced due to PP2CA inhibition [68]. Additionally, 14-3-3 proteins regulate Arabidopsis KAT1, as shown in Xenopus laevis oocyte two-electrode voltage clamp experiments, affecting the activation voltage [69]. G protein GPA1 also regulates the inward K^+^ current in Arabidopsis guard cells under ABA treatment [70]. BAG4 (BCL2-ASSOCIATED ATHANOGENE4) enhances K1 transport via KAT1 interaction, facilitating its plasma-membrane localization [71].

#### 5.2.2. Regulation by Assembly

Both animal and plant Shaker K^+^ channels possess a typical tetrameric structure, resulting in homotetrameric channels with identical subunits and heterotetrameric channels with different subunits from the same family. In plant Shaker K^+^ channels, activity regulation via heteromeric assembly enhances adaptability to stress and fulfills physiological needs. The C-terminal intracellular region of plant Shaker K^+^ channels is believed to play a crucial role in their heteromeric assembly [72].

In Arabidopsis, KAT2 and AKT2 from the Shaker family form heteromeric channels via their alpha subunits, showing stronger binding in yeast two-hybrid β-Gal assays than between their respective alpha subunits. In Xenopus laevis oocytes, KAT2 and AKT2 heteromeric channels display both KAT2’s voltage and time-dependent properties and AtAKT2’s voltage-dependent, time-independent properties. Mutations in the P-loop structure of either subunit can impact both channels’ activity [73]. The Shaker family K^+^ channel AtKC1 interacts with AKT1 [39], with Xenopus laevis oocyte experiments demonstrating that AtKC1-AKT1 heteromerization alters AKT1’s channel properties. This heteromerization results in a negative shift in AKT1’s activation voltage, reducing inward K^+^ current and maximal conductance and inhibiting outward K^+^ flow under 1 mM extracellular K^+^ conditions [44]. Additionally, AtKC1 can form heterotetramers with other Shaker channels (KAT1, KAT2, and AKT2), similarly affecting their half-activation voltages and maximal conductances [41].

#### 5.2.3. Regulation in Protein Localization

Localization regulation is critical for the functional expression of many Shaker K^+^ channel proteins. Currently, two primary types of ion-channel localization regulation are well-understood. One type involves forming hetero-oligomers with Shaker members that have different localization patterns, thereby altering the channel’s activity. For example, AtKC1, localized to the intracellular membrane system, co-localizes with AKT1 to the plasma membrane when co-expressed, forming hetero-oligomers that inhibit the AKT1 current [43]. In guard cells, KAT2 is localized to the cytoplasmic membrane and AKT2 to cytoplasmic vesicles. KAT2 and AKT2 co-express in both the plasma membrane and cytoplasmic vesicles [73]. The second type of localization regulation involves the SNARE family, responsible for vesicle transport. Studies indicate Arabidopsis SYP121 (SYR1/PEN1) facilitates the plasma-membrane transport of ion channels. SYP121 directly interacts with AtKC1 to form complexes with AtKC1 and AKT1, regulating AKT1’s inward K^+^ current characteristics [74]. Additionally, tomato SYP121 crucially regulates the plasma-membrane localization of the guard-cell ion channel KAT1 [75].

#### 5.2.4. Regulation by pH

The channel activity of Shaker K^+^ channels is particularly sensitive to intracellular and extracellular pH regulation due to highly conserved histidine residues in their pore domain. Under different pH conditions, the protonation status of histidine residues can induce conformational changes in the protein, affecting channel opening and closing. Members of the Shaker family, including Arabidopsis KAT2 and SPIK, are activated in acidic extracellular environments. Others, such as Arabidopsis KAT1 and potato KST, are activated in acidic intracellular environments. However, channels like SKOR and AKT2 are inhibited by acidic environments both intracellularly and extracellularly [31,36].

K^+^ channels experience pH regulation differently. For example, pH can affect the current amplitude of the KAT1 [49] and SKOR [31] channels without altering their properties. Conversely, the KAT2 [33], KST1 [76], SPIK [77], AKT2 [31], and ZMK2 [51] channels are pH-sensitive regarding their conductance properties.

#### 5.2.5. Voltage Regulation

**Figure 4:**
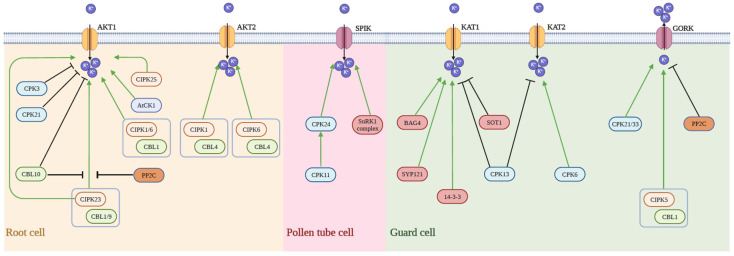
Regulation pathways of some members of the Shaker family.

Plant cells undergo changes in transmembrane potential in response to stimuli, including low K^+^, high salt, mechanical stress, and pathogens [78,79]. Plant Shaker K^+^ channels detect these changes through specialized voltage sensors in their protein structure, regulating channel activity accordingly [80]. Channels variably respond to transmembrane voltage changes, classified as either hyperpolarization-activated or depolarization-activated K^+^ channels. Hyperpolarization-activated channels in Arabidopsis include KAT1, KAT2, AKT1, SPIK, and AKT2, whereas SKOR and GORK are depolarization-activated outward K^+^ channels [38,73]. Although voltage-sensitive structures in the TPK family’s protein structures have not been identified, some members undergo voltage regulation [81]. Currently, the TPK and Kir-like ion-channel families, along with the KUPHAK/KT, HKT, and CPA transporter family members, are not found to be voltage regulated [82] (see Figure 4).

## 6. Strategies and Research Methods for Plant K^+^ Channels

In the study of plant K^+^ channels, researchers utilize a blend of general gene-function research strategies alongside specific techniques and methods tailored for K^+^ channels.

### 6.1. Bioinformatics Prediction and Analysis

Bioinformatics encompasses the acquisition, processing, storage, organization, classification, analysis, and interpretation of biological data. This field is underpinned by high-throughput, large-scale experiments, statistical analyses, and computational tools. In the genomics era, bioinformatics concentrated on sequence assembly, comparison, molecular evolution analysis, protein structure prediction, gene prediction, and non-coding DNA function research. In the post-genomics era, pivotal areas like transcriptome analysis, transcriptomics, metabolic network analysis, and drug-target screening emerged as essential research directions. Bioinformatics primarily targets the study of enzymes, proteins, hormones, and other molecules in plant gene function, evaluating characteristics like sequence features, structural functions, and clustering analysis to uncover gene mechanisms, setting the stage for further detailed research.

Currently, bioinformatics has predicted and analyzed numerous Shaker family members across species, comparing them with classic members from Arabidopsis thaliana and rice. These members typically feature elements, such as the K^+^ channel KAT/AKT, ion transport domain, cyclic nucleotide-binding domain, KHA domain, ankyrin repeats, and voltage-dependent K^+^ channels. Shaker family members have been identified in species including millet [83], soybean [84], sweet potato [85], and cotton [86].

### 6.2. Heterologous Expression System

Heterologous gene expression involves transferring a foreign gene into a target organism to elucidate its biological function, playing a pivotal role in gene-function research. Yeast, African clawed frog oocytes, and Arabidopsis serve as primary host systems for validating plant K^+^ ion-channel studies. This approach facilitates a deeper understanding of the channels’ roles, mechanisms, and contributions to plant physiology and adaptability to environmental stimuli.

#### 6.2.1. Evaluating Yeast Strains for Low K^+^ Tolerance through Genetic Expression Analysis

Using yeast mutants to validate heterologous gene functions for specific traits or isolating genes through library screening provides an efficient method for gene cloning and functional validation in higher eukaryotes. The Saccharomyces cerevisiae and methanotrophic yeast systems are the most commonly used yeast expression systems. Yeast, a single-celled lower eukaryote, flourishes under standard culture conditions with rapid growth, high tolerance, and low production costs. It is safe, non-toxic, and streamlines molecular biology procedures. When integrated into yeast chromosomes with expression vectors, foreign genes are replicated and inherited reliably.

Yeast mutants deficient in K^+^ cannot grow in low-K^+^ environments. However, introducing an exogenous cDNA or cDNA library cloned onto an expression vector into these mutants can restore their growth. K^+^ absorption-deficient yeast facilitated the identification of the first plant Shaker K^+^ channel, AKT1, in Arabidopsis thaliana [13]. Subsequently, AKT1 in rice [87], maize’s KZM2 (similar to Arabidopsis KAT1) [88], and cassava’s AKT2 [89] were identified. Additionally, rice AKT1, discovered using salt-sensitive yeast, underscores its role in salt tolerance [90].

#### 6.2.2. Physiological Assessment of Heterologous K^+^ Channel Expression in Xenopus Oocytes

Xenopus laevis oocytes are widely used for the heterologous expression, identification, and regulation of biological ion channels. Injection of exogenous ion-channel genes facilitates the expression of active channel proteins, enabling functional characterization and electrophysiological analysis [91]. Advantages of Xenopus laevis oocytes include ease of material procurement, no stringent requirement for sterile cell-culture environments or expensive culture solutions, and a large size that enables single-cell-level operations. As a transient expression system, it provides rapid, stable, and comparative means to identify channel proteins and study regulatory mechanisms.

However, the system has limitations. Expressed exogenous proteins may be influenced by the oocyte’s endogenous channels, potentially affecting the expression and functional identification of these proteins. Experimental outcomes may vary based on oocyte conditions (batch, season, temperature, etc.), and the subcellular localization of exogenous proteins may differ from in vivo conditions, affecting experimental reliability [92].

#### 6.2.3. Model Plant Expression and Phenotypic Identification

Introducing the target gene into Arabidopsis thaliana is a commonly employed research strategy. Compared to yeast and Xenopus oocytes, Arabidopsis thaliana provides a genetic background closer to the plant’s, facilitating an intuitive assessment of the gene’s functional impact. Arabidopsis thaliana, a model organism in molecular biology, offers advantages like a short growth cycle, a fully sequenced genome, controllable growth conditions, and efficient transformation methods.

However, it has limitations, including limited practical applications, dependence on specific light conditions, and notable genetic susceptibility. OsAKT1 and ZMK1, sharing K^+^ absorption functions and phenotypes with AtAKT1, were first validated functionally in Arabidopsis [87,93]. This approach allows researchers to closely examine the gene’s role within a plant system, enhancing our understanding of its physiological relevance and potential contributions to plant biology and agriculture.

### 6.3. Detection of K^+^ Channel Activity by Electrophysiological Technology

Electrophysiological technology involves stimulating living organisms with various stimuli (light, electricity, pressure, chemical substances, etc.) to measure, record, and analyze bioelectric phenomena and electrical characteristics. This method is essential for detecting K^+^ channel activity. The two-electrode voltage clamp and patch-clamp techniques are extensively employed for K^+^ channel identification. These techniques provide detailed insights into the functionality and regulation of K^+^ channels, contributing significantly to our understanding of their roles in cellular and physiological processes.

#### 6.3.1. Two-Electrode Voltage Clamp Technology

The two-electrode voltage clamp technique, rooted in electrophysiology, precisely controls the target cell membrane’s potential and measures ion-channel currents in real time, facilitating the study of channel function and dynamics. It is commonly used to investigate ion channels, like K^+^ channels, and their responses to specific potentials. Adjusting V_cmd sets the cell membrane potential, and the current through the ion channel at a specific voltage level is measured to analyze its characteristics.

The Xenopus oocyte heterologous expression system combined with two-electrode voltage clamp technology is extensively used for plant channel identification and regulatory research. KAT1 was the first Arabidopsis K^+^ channel identified using this system [13]. Since then, a series of plant ion channels have been identified in Xenopus laevis oocytes, including Arabidopsis SKOR [45], AKT2 [68], GORK [46], faba bean VFK1 [94], and maize ZmK2.1 [95].

**Figure 5:**
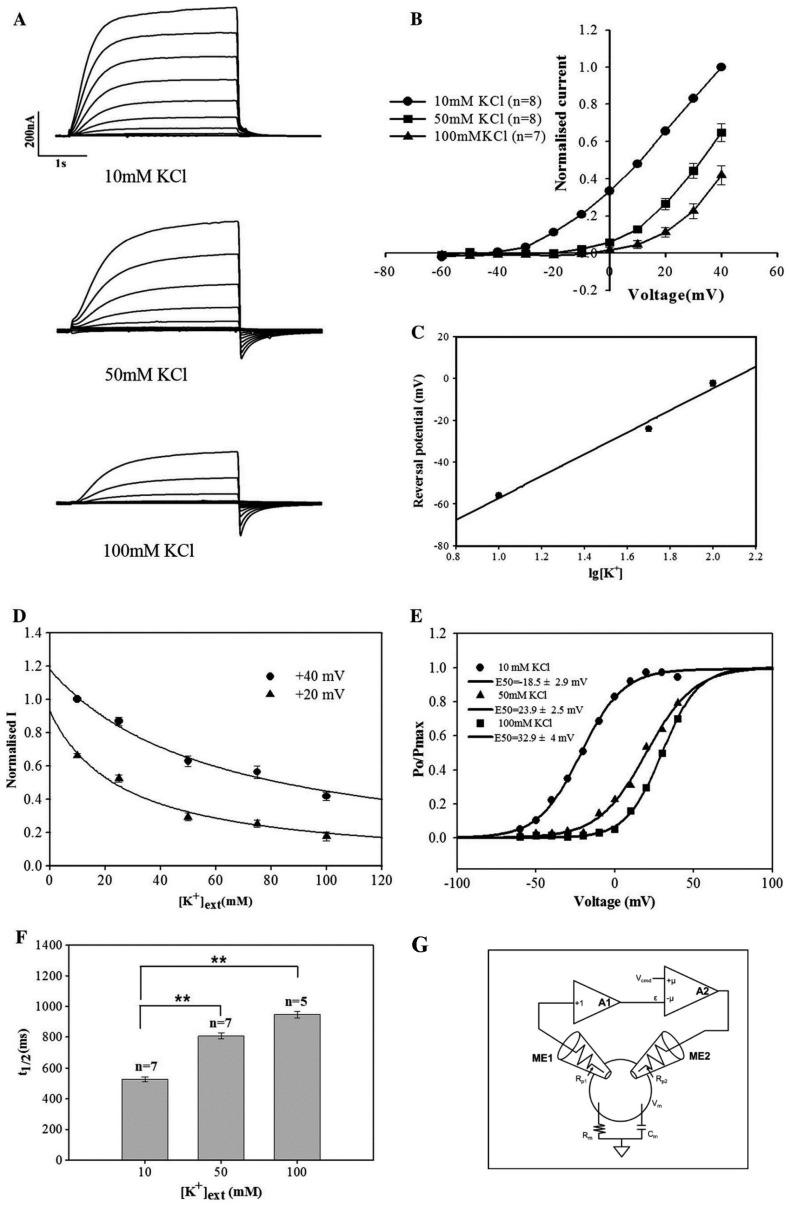
Analysis of two-electrode voltage clamp experiments and overview of patch-clamp technique principles. (**A**) Utilizing different external KCl concentrations (10 mM, 50 mM, and 100 mM), a series of depolarizing voltage pulses were applied to oocyte cells. The resultant current waveforms recorded under these conditions provided insights into the channels’ responsiveness to depolarization. (**B**) The data obtained were plotted to represent the current–voltage (I–V) relationship curve, crucial for understanding the conductive properties of the ion channels across different voltage levels. (**C**) A notable observation was the linear relationship between the reversal potential and the logarithm of external K^+^ concentration, elucidating the dependency of ion-channel activity on external K^+^ levels. (**D**) An increase in external K^+^ concentration led to an enhanced inhibitory effect on the current, delineating a reverse saturation kinetics curve. This phenomenon underscores the channels’ regulatory mechanisms in response to K^+^ concentration changes. (**E**) The activation (opening) curve of the channels, indicated by the half-maximal activation potential (E50), exhibited a positive shift with the increase of external K^+^ concentrations. This shift highlights the modulation of channel-activation thresholds by external K^+^ levels. (**F**) Bar graphs were employed to illustrate the variations in activation half time (t1/2) with changing external K^+^ concentrations. The activation half time, indicative of the duration required for channels to transition from closed to open states, demonstrated a deceleration in channel opening rates as external K^+^ concentrations elevated. Results were shown as mean ± SE. Unpaired Student’s *t*-tests were applied to assess significance. ** *p* < 0.01 (**G**) The schematic diagram of the dual-electrode voltage clamp setup elucidates the experimental framework. Microelectrodes (ME1 and ME2) measure cell membrane potential (Vm) and facilitate current injection. The voltage command (V_cmd) maintains the desired membrane potential, with amplifiers (A1 and A2) enhancing signal detection. A1 monitors the potential difference (Vm) across the electrodes, while A2 assesses discrepancies between V_cmd and the actual membrane potential (ε), modulating the current (±μ) accordingly. Feedback resistors (Rp1 and Rp2) adjust the membrane potential to desired levels, with membrane resistance (Rm) and capacitance (Cm) reflecting ion-channel resistance and the membrane’s charge storage capacity, respectively.

Taking the outward K^+^ channel GORK from the Mongolian Shadong Shaker family as an example, we briefly describe its functional analysis using the two-electrode voltage clamp technique [96]. When external K^+^ is present, the depolarizing voltage applied to the oocyte membrane induces an outward current in AmGORK-injected oocytes. Increasing the external K^+^ concentration leads to a gradual decrease in current amplitude, indicating that AmGORK is an outward K^+^ channel with release dependent on the external K^+^ concentration. A tenfold increase in external K^+^ concentration shifts AmGORK’s reversal potential Vrev by 52.4 ± 2.3 mV, suggesting primary K^+^ conduction. The AmGORK current inhibition displays reverse saturation kinetics related to K^+^ concentration. Further analysis was conducted on AmGORK’s gating properties and its response to membrane voltage and external K^+^ concentration. The activation half-life of AmGORK in the presence of 10 mM, 50 mM, and 100 mM K^+^ was, respectively, 526.7 ± 16.6 ms, 806 ± 20 ms, and 947 ± 21.5 ms. Thus, AmGORK-mediated K^+^ release is dependent on both voltage and external K^+^ concentration (see Figure 5).

#### 6.3.2. Patch-Clamp Technique

The patch-clamp technique, a highly precise electrophysiological method, records the current activity of single or multiple ion channels on the cell membrane. This technique achieves high-resolution current recordings by forming a high-resistance gigaseal and offers four basic recording modes: cell attached, whole cell, intracellular, and extracellular. Compared to the voltage clamp technique, the patch clamp offers higher-resolution current recordings, enabling precise control over the cell membrane potential and direct drug application to study ion-channel effects. However, this method requires significant technical skill and specific experimental conditions, may damage cells, and involves complex data processing.

In plant-cell ion-channel studies, patch-clamp technology is primarily used to record transmembrane ion currents and analyze ion-channel characteristics. Protoplasts are prepared by removing the cell walls. This method has been successfully applied to various plant-cell types. Researchers have identified channels, such as the Shaker channel AKT in root cells [29], the Shaker K^+^ channel SPIK in pollen [30], and the outward K^+^ channel GORK and anion channel SLAC1 in guard cells [46,47].

**Figure 6:**
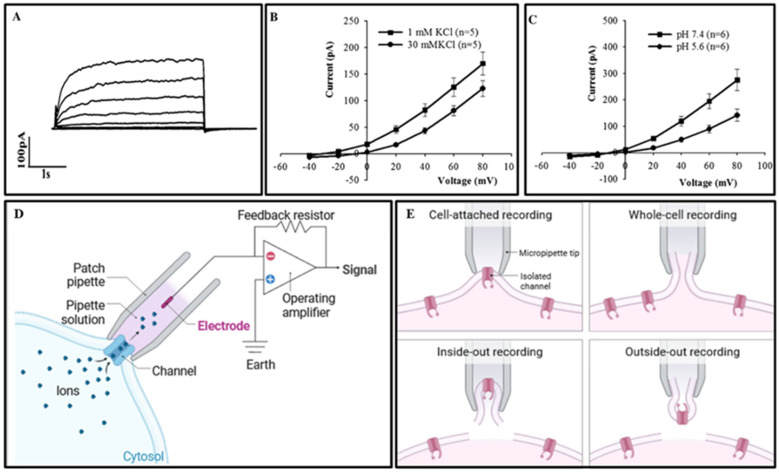
Analysis of patch-clamp results and schematic diagram of the principle model. (**A**) In this set of experiments, whole-cell recordings were obtained for outward K⁺ currents. Mongolicus guard-cell protoplasts immersed in a bath solution containing 1 mM KCl. The recordings were initiated through voltage pulses increasing by 20 mV from −40 mV to +80 mV, systematically activating K⁺_out currents. This procedure helped delineate the average current–voltage (I-V) relationships, assessing the ion channels’ response to incremental K⁺ concentrations. (**B**) The experiment demonstrated the ion channels’ responsiveness to alterations in K⁺ concentration within the external medium, observed by transitioning from 1 mM (represented as solid squares) to 30 mM (shown as solid circles). This shift illustrates the channels’ functional dynamics in varying K⁺ environments. (**C**) Adjustments in external pH values, from 7.4 (solid squares) to 5.6 (solid circles), also significantly impacted the ion-channel activity, underscoring the sensitivity of K⁺ currents to pH fluctuations in the surrounding environment. (**D**) This segment details the fundamental working principle of the patch-clamp technique, a sophisticated electrophysiological method for recording the electrical currents passing through individual or multiple ion channels with high fidelity. (**E**) Describes the four core measurement modes employed in patch-clamp studies: cell-attached, whole-cell, inside-out, and outside-out configurations. Each mode offers unique insights into the ion channels’ operational mechanics, from single-channel activities to the collective behavior of channels across the entire cell membrane.

Taking GORK, the outward-directed K^+^ channel from the Mongolian Shadon shaker family, as an example, we will briefly describe its identification and analysis using whole-cell patch-clamp technology [96]. At depolarized membrane potentials, a typical bulk K^+^ outward current is observed in a voltage- and time-dependent manner (Figure 6A). Furthermore, increasing the external K^+^ concentration from 1 mM to 30 mM (resulting in a 47% reduction at a membrane potential of ^+^40 mV) and altering the external pH from 7.4 to 5.6 (leading to a 63% reduction at +40 mV) decreased the outward rectifying current (Figure 6B,C), indicating that guard-cell K^+^ efflux depends on voltage and K^+^ concentration, and is inhibited by extracellular acidification (see Figure 6).

## 7. Prospectives

Investigating Shaker-type K^+^ channels in plants holds profound significance for molecular biology. These channels play a critical role in regulating plant growth and development, enriching our understanding of their regulatory mechanisms. Additionally, they modulate intracellular K^+^ ion concentrations in response to environmental stresses, such as drought, salinity, and low temperatures. This function is crucial for maintaining the cell membrane’s potential stability and cellular activities, which contributes to enhancing crop resilience and K^+^ utilization efficiency—essential for global agricultural sustainability.

Despite significant breakthroughs, ion-channel research still requires in-depth exploration in many areas. Advancing our understanding of ion-channel selectivity, dynamics, and regulatory mechanisms necessitates acquiring higher-resolution protein structures. Consequently, developing structural biology techniques specifically for plant studies is urgently needed. The diverse and overlapping functions of Shaker channel members require comprehensive analysis across genetics, biochemistry, and biophysics. Furthermore, a thorough investigation of Shaker channels’ regulatory mechanisms necessitates considering complex cell signaling networks. This approach will enable a deeper understanding of their critical roles in plant growth and stress responses.

Extensive genomics and bioinformatics research is crucial for unveiling the full spectrum of the plant Shaker channel family. Translating research findings into practical applications is essential to fully leverage this research domain’s potential. In summary, deep research into Shaker-type K^+^ ion channels in plants enhances our understanding of plant biology and offers strategies to boost agricultural productivity and address global food-security challenges. Overcoming the hurdles in structural and functional analysis demands ongoing efforts and interdisciplinary collaboration.

## Figures and Tables

**Table 1:** Characteristics of different K^+^ channels.

Gene Family Name	Shaker	TPK	Kir-like	CNGC
Topological structure	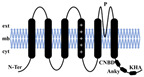	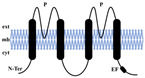	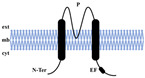	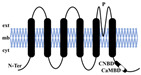
Structural characteristics	TM1-6 1 Pore	TM1-4 2 Pores	TM1-2 1 Pore	TM1-6 1 Pore
Subgroup divisions and representative members	Ⅰ: AKT1, AKT5, AKT6 Ⅱ:KAT1, KAT2 Ⅲ: AKT2 Ⅳ: AtKC1 Ⅴ: GORK, SKOR	AtTPK1, AtTPK2, AtTPK3, AtTPK4, AtTPK5,	AtKCO3	AtCNGC1 AtCNGC2
